# Machine learning identifies a strong association between warming and reduced primary productivity in an oligotrophic ocean gyre

**DOI:** 10.1038/s41598-020-59989-y

**Published:** 2020-02-25

**Authors:** Domenico D’Alelio, Salvatore Rampone, Luigi Maria Cusano, Valerio Morfino, Luca Russo, Nadia Sanseverino, James E. Cloern, Michael W. Lomas

**Affiliations:** 1 Department of Integrative Marine Ecology, Stazione Zoologica Anton Dohrn, Villa Comunale, I-80121 Naples, Italy; 2 Università degli Studi del Sannio, Via Delle Puglie 76, I-82100 Benevento, Italy; 3 United States Geological Survey (emeritus), Menlo Park, CA USA; 4 Bigelow Laboratory for Ocean Sciences, East Boothbay, ME USA

**Keywords:** Microbial ecology, Marine biology, Applied mathematics

## Abstract

Phytoplankton play key roles in the oceans by regulating global biogeochemical cycles and production in marine food webs. Global warming is thought to affect phytoplankton production both directly, by impacting their photosynthetic metabolism, and indirectly by modifying the physical environment in which they grow. In this respect, the Bermuda Atlantic Time-series Study (BATS) in the Sargasso Sea (North Atlantic gyre) provides a unique opportunity to explore effects of warming on phytoplankton production across the vast oligotrophic ocean regions because it is one of the few multidecadal records of measured net primary productivity (NPP). We analysed the time series of phytoplankton primary productivity at BATS site using machine learning techniques (ML) to show that increased water temperature over a 27-year period (1990–2016), and the consequent weakening of vertical mixing in the upper ocean, induced a negative feedback on phytoplankton productivity by reducing the availability of essential resources, nitrogen and light. The unbalanced availability of these resources with warming, coupled with ecological changes at the community level, is expected to intensify the oligotrophic state of open-ocean regions that are far from land-based nutrient sources.

## Introduction

Phytoplankton play key roles in the oceans by regulating global biogeochemical cycles and production in marine food webs^1,2^. Global warming is thought to affect phytoplankton production both directly, by impacting photosynthetic metabolism^3^, and indirectly by modifying the physical environment^4^. Interest in the impact of global warming on phytoplankton has grown during the last decade^5^, with observations of synchronous increases of surface ocean temperature and an apparent global decrease of phytoplankton biomass, primarily inferred from changes in chlorophyll *a* (Chl *a*) concentration^6,7^.

However, the conclusion about a decrease of phytoplankton across the last century is weakly supported because of the paucity of data before the 1980s^8^, and because some observational studies have shown an opposite trend while others revealed that Chl *a* declines were partly due to photo-acclimation: other photosynthetic pigments rose to dominance through changes at the cellular level and alternations between different phytoplankton groups^9,10^. Thus, the relationship between ocean physics and phytoplankton blooms can be mediated by a number of ecological factors, such as inter-group competition, which confound our understanding of long-term productivity trends^11,12^.

Most studies on the relation between primary production and global change have been grounded in observations of phytoplankton biomass, rather than direct measurements of primary productivity. However, phytoplankton biomass is determined by multiple sources and sinks and a myriad of mortality factors (e.g., grazing by zooplankton and infection by parasites)^13^. As a result, changes in phytoplankton biomass over time do not necessarily parallel changes in the rate of primary production, i.e. the primary productivity, which reflects the level of photosynthetic activity of planktonic microalgae and is strongly linked to the availability of primary resources, such as nutrients and light energy.

From this conceptual basis, global warming is thought to reduce vertical mixing in the oceans and its transport of nutrients from deep waters to the photic zone. As a result, primary productivity is expected to decrease in a warming ocean. Although empirical evidence is not sufficiently robust to fully validate this concept, several model examples yield results that are consistent with it^14,15^. A main limitation of these models is that they are based on phytoplankton data taken from the ocean surface. Further validations are possible with long-term data representative of changes over the photic-zone depth that extends well below the surface in oligotrophic oceans^16^.

**Figure 1:**
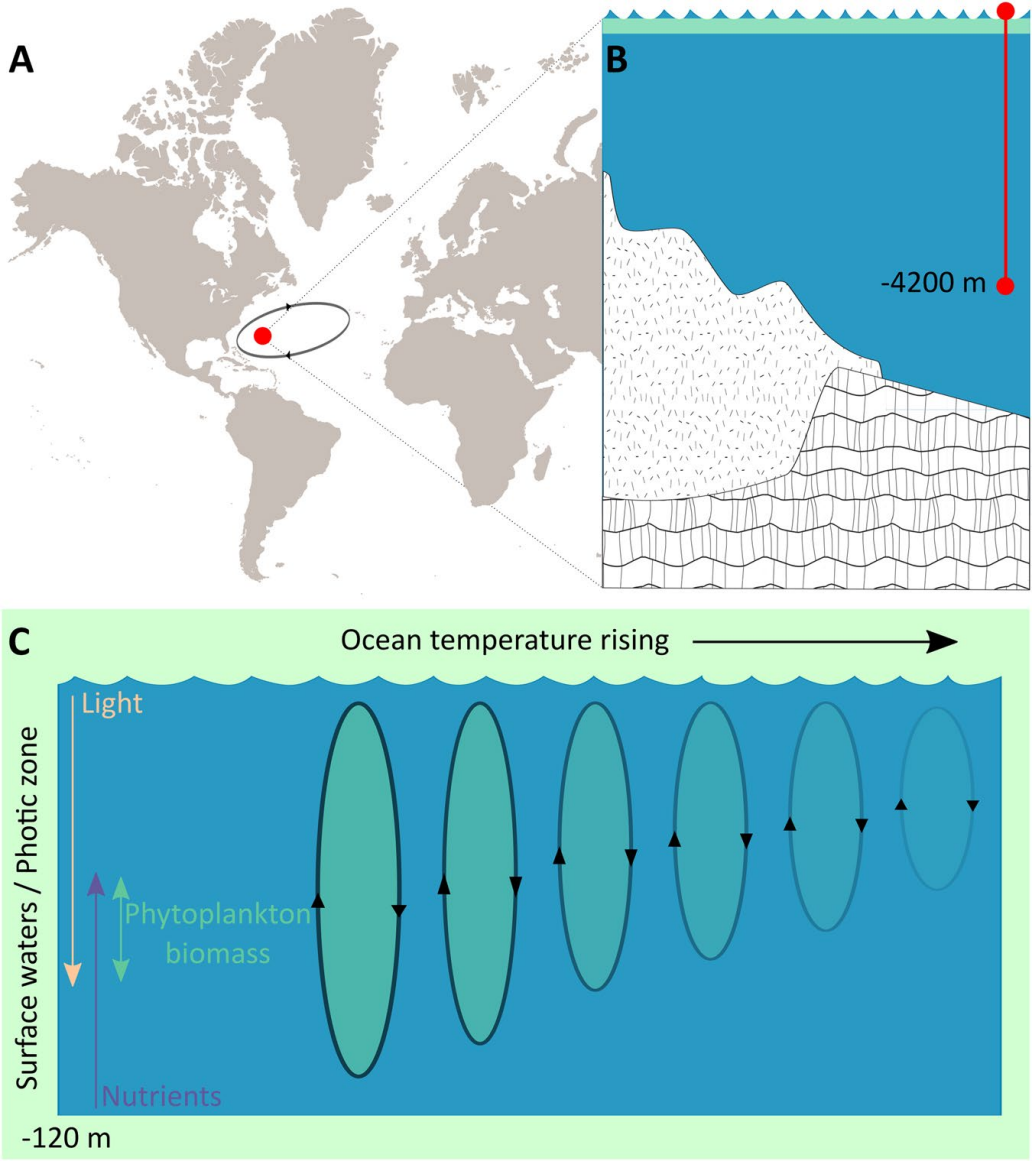
Study system and working hypothesis: productivity changes at the BATS site. (**A**) Geographic location of the BATS time series site in the western boundary of the North Atlantic gyre. (**B**) Vertical extension of the sampling site, in comparison with the shelf profile (the surface water layer is shown in green). (**C**) Factors that potentially affect primary production and a conceptual scheme for the decrease of phytoplankton biomass in the surface waters as warming proceeds.

Stratification is classically considered as a main mechanism controlling variability in ocean productivity because it determines the availability of light and nutrients^17^, especially in those sectors far from continental shelves where surface water cannot be replenished of nutrients from land runoff^18^. One such region is the Sargasso Sea, which hosts the Bermuda Atlantic Time-series Study (BATS) that has measured primary productivity over a period of multiple decades^16^ (Fig. 1). The BATS series provides a unique opportunity to explore effects of warming and, thus, reduced stratification, on primary productivity across the vast oligotrophic ocean regions because it is one of the few multidecadal records of measured net primary productivity (NPP).

In this paper, we analysed an oceanographic dataset from BATS that represents variability over the entire photic zone (upper 120 m depth) to search for mechanistic links between primary productivity, vertical mixing, and availability of nutrient and light resources. We analysed a time series of phytoplankton primary productivity over a 27-year period (1990–2016) in comparison with physical-chemical data, using both linear statistics and machine learning techniques (ML). Finally, we discuss the ecological implication of changes in phytoplankton activity potentially driven by global warming.

## Physical-Ecological Context and Study Design

The Bermuda Atlantic Time-series Study is a biogeochemical time-series with a focus on how plankton impact the cycling of carbon and other biogeochemically relevant elements. The BATS station is located in the north western quadrant of the Sargasso Sea, 82 km south east of Bermuda (31°40′N, 64°10′W), where monthly sampling (fortnightly during the winter/spring mixing period) began in October of 1988^19^. The maximum depth is ~4680 m in this area, a vast and deep region of the subtropical sector of the North Atlantic.

The Sargasso Sea is isolated from other basins by an ocean gyre, a clockwise oceanic circulation extending from the Gulf of Mexico to the Azores (e.g.^20,21^). This condition isolates the BATS site from terrigenous influences originating from the East Coast of the United States (Fig. 1). Thus, the upper Sargasso Sea has oligotrophic characteristics, with nutrient concentrations at or below the detection threshold with standard methods. For instance, the highest nitrate and phosphate concentrations are detected below 80–100 m and 150–100 m, respectively^19^.

As a consequence, the clear water condition results in a deep euphotic zone of roughly 100 m, so phytoplankton primary production is highly dependent on events of deepened vertical circulation that transport nutrients to the surface. These environmental characteristics make BATS site a valuable case-study for investigating relationships between phytoplankton productivity, water stratification and mixing, as suggested by previous observations of both year and year-over-year variabilities at that site^16^ (Fig. 1).

**Figure 2:**
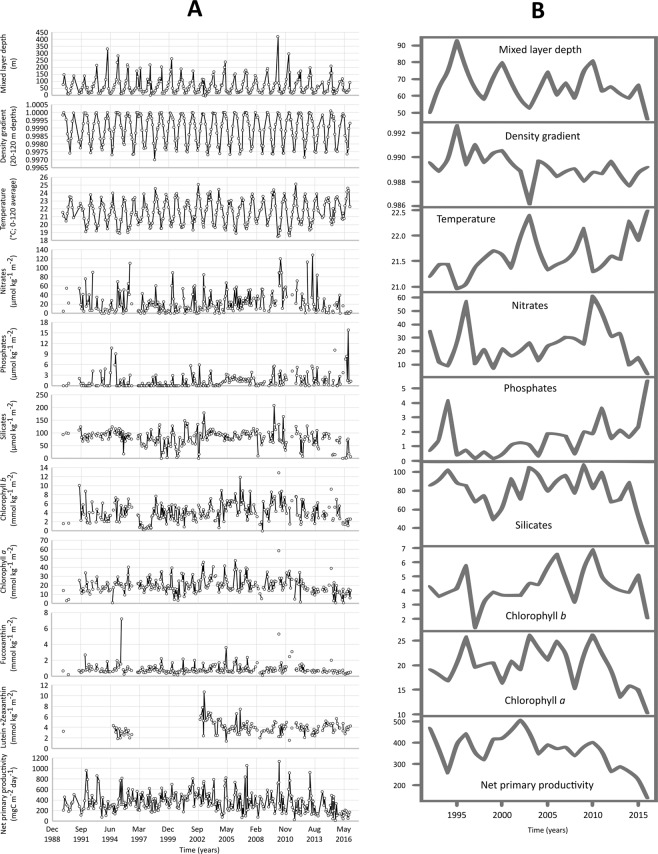
Long term series at the BATS station. (**A**) Time series for the environmental and biological variables investigated herein. (**B**) Time series of annual means from 1990 to 2016; units in the y-axis are the same as in (**A**).

From the BATS record, we constructed a 1990–2016 time series from measurements made in the photic zone (0–120 m). The time series included measurements of: (i) net phytoplankton productivity (integrated between 0 and 120 m); (ii) water temperature (0–120 m mean); (iii) degree of stratification (as the ratio between densities at 20 and 120 m); (iv) depth of the mixed layer; and (v) concentrations of macronutrients (i.e., NO_3_, PO_4_, SiO_2_,) and four photosynthetic pigments (i.e., chlorophyll *a* and *b*, fucoxanthin, and lutein plus zeaxanthin), all integrated over the upper 120 m (Fig. 2A). We explored changes in these variables over time, with a focus on interannual variability, with traditional and more advanced (machine learning) statistics (detailed procedures are provided in the Methods).

We first explored long-term trends over the full time series by applying the nonparametric Seasonal Kendall test to physical/chemical/biological data aggregated by month. This step revealed the presence or absence of significant changes over the full record, and identified possible associations between variables based on their co-variance in time. Next, we applied the same trend test on sequential 10-year windows of the data series to identify decadal patterns of change that occurred within the complete series. This step is useful, for example, in identifying oscillations within a series having a long-term trend^22^.

Guided by these analyses, we then applied linear and non-linear machine-learning (ML) tools to the physical-chemical-biological dataset over the full record to: (i) test the associations between variables with more robust mathematical methods, (ii) identify the mathematical laws (i.e. equations, say, functions) that define interrelations between variables, and (iii) search for possible mechanisms underlying the observed patterns.

In general terms, we employed the so-called supervised machine learning, suitable for time series forecasting^23^, in which an algorithm ‘learns’ to derive the mapping function between a number of input variables (x) and one output variable (y). The goal is to approximate the real underlying mapping so well that, using the final function, it is possible to use new input variables to predict the unknown output variable. In our specific case, the net primary productivity was the output variable.

Among ML techniques applied herein, the Genetic Programming^24^ was the most sophisticated one: it is based on ‘evolving’ algorithms^25^, which generate and evolve unknown functions automatically, usually represented as tree structures, which can both mutate and reciprocally recombine, as it happens with the evolving DNA^26^. The final equation of each ML experiment was tested with a sensitivity analysis that allowed us to identify the differential impact exerted by all the potential input variables on NPP.

## Results and Discussion

### Temperature and productivity trends

**Figure 3:**
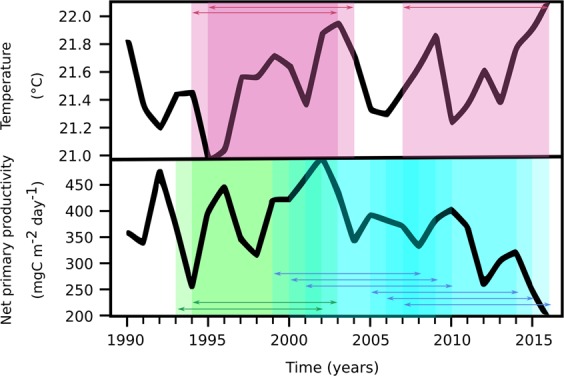
Associated trends of temperature and net primary productivity at the BATS site. The annual averaged time-series of temperature and net primary productivity (NPP) are shown, in comparison with the results of time-windowed Seasonal Kendall tests (window = 10 years) conducted on the monthly-averaged time series. In the uppermost plot, red boxes highlight periods of significant warming; in the lowermost plot, green and blue boxes highlight periods of significant increasing and decreasing of NPP, respectively. In both plots, arrows indicate overlapping between subsequent windows showing statistically significant ten-years trends.

The analysis of the whole dataset, employing traditional statistics on the monthly series, is shown in Figs. 2, 3 and Tables S1 and 2. Along with raw data (Fig. 2A), we also show the long-term series of annually averaged data (Fig. 2B). This record showed large interannual variability of all variables, revealing patterns more complex than simple and synchronous monotonic changes in time. Trend analysis identified highly significant long-term changes in only two variables as: temperature increase and NPP decrease (p < 0.001; Table S1). Phosphate concentration also showed a positive trend that was statistically significant (p < 0.001), but this result was less reliable because it was driven by one isolated spike in the dataset at the end of the series. Slightly significant trends (p < 0.05) were detected for concentrations of silicate (negative trend) and chlorophyll *b* (positive trend). No significant trend was identified for the mixed-layer depth or the density gradient.

As further evidence of the putative association between temperature and NPP, the time-windowed analysis identified an opposite covariance of these variables over the whole record (Fig. 3). Positive trends in temperature were detected in the periods 1994–2004 (p < 0.01) and 2007–2016 (p = 0.02), with the first being the decade of fastest warming. Positive trends of NPP were detected over the period 1993–2003 (p < 0.01), in agreement with analyses of Saba and co-authors^27^ and Lomas *et al*.^16^ who reported a 2% increase per year in NPP from 1989 to 2007. However, our analysis of the 1990–2016 record identified two eras of NPP decrease after 2007: 1999–2010 (p = 0.01), and 2005–2016 (p < 0.01), with the fastest decrease occurring between 2007 and 2016. These results suggest the possibility of a biogeochemical transition at the BATS site beginning in the mid-2000s.

Based on our working hypothesis, the overall negative NPP trend could be related to the long-term changes in the physical properties of the water column, driven by the multi-decadal rising of temperature in the photic zone. However, as revealed by the time-windowed analysis, the fastest decade of decreased NPP occurred at the end of the record, while the fastest temperature increase occurred from 1994–2004. Thus, even though 2010–2016 was the period of highest heat accumulation in the upper (<−700 m depth) Atlantic Ocean over the last 150 years^28^, we cannot attribute the largest drops in NPP (occurring in the last decade of the time series) solely to warming because there was an earlier decade when temperature increased at a faster rate. One possible explanation for this observation is that the fast warming between 1995 and 2004 induced a a physiological response in phytoplankton (e.g., changing C:P ratios in cells) that maintained high NPP levels, whereas the following further warming led to conditions outside the cells’ physiological range and, thus, the collapse of NPP. However, as a general consideration, mechanistic interpretations should be limited at this stage because of potential inter-dependencies of several variables that can affect NPP.

### Mechanisms behind productivity trend

Ocean monitoring is increasingly applied to use observed dynamics of physical-chemical variables to hindcast, and eventually forecast, the dynamics of key processes such as primary productivity. However, oceanographic databases often include sparse and missing values of variables having large-amplitude fluctuations and high variance. As a result, these datasets contain weak signals when explored with traditional statistical techniques, which basically work under the assumption of linearity. In fact, ecological processes are driven by nonlinear responses to perturbation^29,30^, such as changes in community composition, physiological acclimation and evolutionary adaptation to change, which can be fast and dramatic in phytoplankton^31^. Moreover, due to the complexity of plankton communities, environmental factors can affect key functional groups both directly and indirectly, e.g. through indirect effects playing at community level^32^, and long-term amplifications occurring at ecosystem scale^33^.

To explore mechanistic relationships between phytoplankton productivity, the chemical environment, and stratification dynamics, and to identify the mathematical laws mapping those relationships, we applied different categories of ML techniques to the BATS dataset. Results from ML are summarized in Table 1, showing correlation coefficients between measured and mathematically-predicted NPP and the Mean Absolute Error (MAE) as a metric of model skill^34^. Among all techniques, the best match (highest correlation, smallest MAE) between measured and predicted NPP was obtained from Genetic Programming (GP), and we discuss herein the outcome of that analysis in detail. Nonetheless, we must note that the best performing ML method still has a 25% error. This reflects the complexity of oceanic systems, including many other potential driving factors that are missed from our analysis, such as biological interactions and algal physiological plasticity that can have cascading effects on ocean biogeochemistry^33,35–37^.

**Table 1:** Statistical tests applied to BATS dataset (machine learning).

Method	Correlation coefficient	Mean absolute error
Gaussian Processes (Linear Kernel)	0.65	74%
Linear Regression Model	0.64	73%
Multilayer Perceptron (Automatic)	0.28	122%
Random Forest	0.523	81%
Support Vector Machine	0.63	77%
Multilayer Perceptron (Manual)	0.72	43%
Genetic Programming	0.70	25%

Machine learning techniques utilized in the present study and applied to the BATS dataset (See Methods).

**Figure 4:**
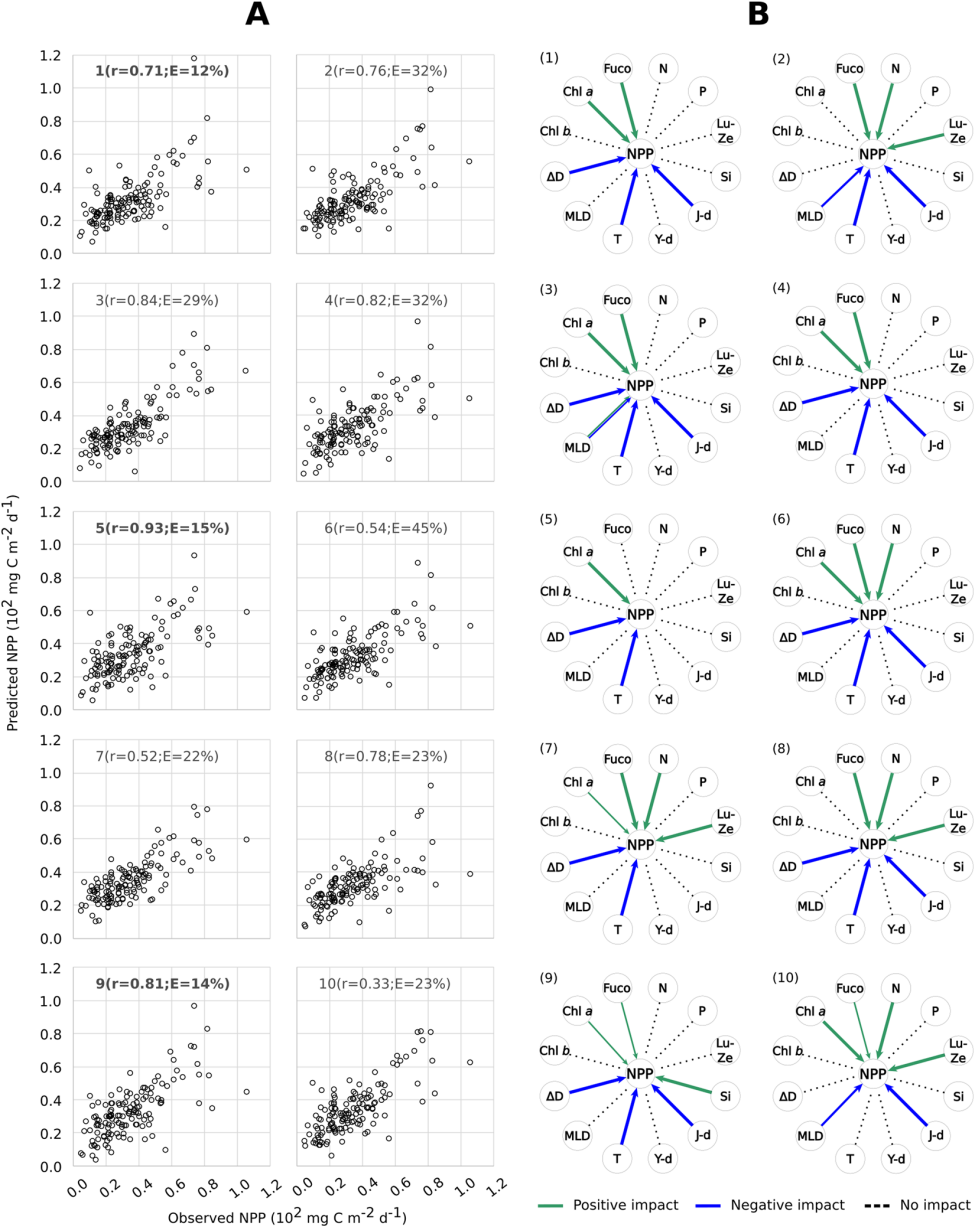
Results of genetic programming analyses on the BATS dataset. (**A**) Comparisons between observed values of net primary productivity (NPP) and those predicted by ten different GP experiments; the regression coefficient ‘r’ and the Mean Absolute Error ‘E’ are indicated for each experiment. The Mean Absolute Error in Table 1 is the average of values indicated in this figure. The experiments with the best match between real and predicted data are in bold. (**B**) Synthetic representation of the impact of different variables (N = nitrates; P = phosphates; Lut-Zea = lutein-zeaxanthin; Si = silicates; J-day = Julian day; Y-day = the day of the year, i.e. a parameter used to express the progress of seasons; T = temperature; MLD = mixed layer depth, ΔD = density gradient; Chl *b* = chlorophyll *b*; Chl *a* = chlorophyll *a*; Fuco = fucoxanthin) on net primary productivity NPP, according to ten GP experiments, coded as (1–10) in the graphs; green and blue arrows indicate either positive or negative impacts of a variable on NPP, respectively; arrow thickness is proportional to the magnitude of the impact.

The match between observed and predicted NPP by the ten different GP experiments is shown in Fig. 4 (see also equations in Methods). Most equations from ML experiments were successful at predicting NPP based on inputs of the physical/chemical/biological variables we considered. In seven out of ten experiments, the correlation coefficient between predicted and measured NPP was higher than 0.7 (Fig. 4A). As a main driving factor, temperature negatively impacted NPP, as it showed a minus sign in the equations derived in nine of the ten experiments (Fig. 4B, Tables S4–13). When considering the sensitivity analysis, deriving from a 10-fold validation procedure performed for each experiment, the negative impact of temperature on NPP was 100% probable in each experiment. This observation verifies the strong dependence of NPP variability on temperature at the BATS station over the 1990–2016 observation period, consistent with the long-term trend analysis above. However, unlike trend analysis, ML allowed us to analyse the impact of other, potentially interconnected variables on net primary productivity that did not show long-term trends.

For instance, the density gradient negatively impacted NPP in eight out of ten experiments and this impact was 100% probable, based on 10-fold validation (Fig. 4B, Tables S4–13). This means that, over the whole period of observation, the smallest NPP coincided with the highest density differences between the surface and deep layers. This association reinforces the hypothesis that NPP decreased between mid-2000s and 2016 due to the parallel stabilization of the water column as stratification intensified. However, we must note that the depth of the mixed layer (MLD) did not show the same influence on NPP as the density gradient. After ML, MLD was present in the final equation in only three out of ten experiments and in two of them it showed a significantly negative impact (see experiments #2, 10; yet, in exp. #3 MLD could impact NPP both positively and negatively with the same 50% probability; Fig. 4B, Tables S4–13). The apparent disconnect between the density gradient and the mixed-layer depth can be explained as follows.

Phytoplankton productivity in the open ocean is regulated by the transport of nutrient-rich deep waters to the photic zone. This transport is mediated by vertical mixing that is damped by thermal stratification^13^. Based on our analyses, NPP is negatively impacted by surface warming and its strengthening of stratification, measured as the density gradient. GP results indicate that MLD had little effect on NPP, suggesting that other factors, such as the rate of mixing and not its absolute vertical extension, are more determinant in the production dynamics^17^. For example, if mixing is slow enough, due to a stronger density gradient, nutrients in the photic zone are exhausted more quickly, thus limiting phytoplankton productivity.

Analogous to the density gradient, Julian day was also well represented in the final equations derived from GP: this variable was included in the outcome of all ten experiments and exerted a strong and negative impact (probability = 100%) over NPP in eight of them (Fig. 4B, Tables S4–13). In this respect, GP confirms the long-term decrease of NPP – i.e., temperature increases and NPP decreases as the observations extend over time.

On the contrary, the day of the year (Y-day) – i.e. an independent variable representing seasons in the mathematical equation from GP – showed no effect on NPP. This result could stem from the fact that the codification of the Y-day from 1 to 365 is not adequate, because it could fail to convey the cyclical character of the seasonal changes, unless it is transformed using periodic functions (e.g. Gregor *et al*.^38^). We addressed this point by running GP with transformed Y-day data, but this operation yielded lower predictive power: in this way Y-day had a forced proportionality with temperature, resulting in a weak correlation between measured and predicted NPP (MAE > 300%). As a matter of fact, formulas deriving from genetic programming (see the ten GP equations in the methods section) identified no role played by annual periodicity in driving the long-term trend of primary productivity. The trend of decreasing NPP was mainly associated with interannual and decadal variability of controlling factors, as already indicated by our trend analysis using linear statistics.

In synthesis, based on GP analysis, temperature, stratification and time (expressed as Julian day) were highly statistically interrelated – i.e., they all showed negative associations in the mathematical formulation of NPP as a function of all the other variables (see GP equations in Methods). These three variables exerted a similar impact on NPP (see sensitivity analyses results in Tables S4–13).

Unlike temperature and stratification, the concentrations of pigments Chl *a* and fucoxanthin exerted a positive impact on NPP, with Chl *a* showing a 100% sensitivity in six out of ten experiments, and fucoxanthin in seven out of ten (Fig. 4B; see also equations in Methods and sensitivity analyses results in Tables S4–13). This suggests that the largest increments of phytoplankton productivity in the Sargasso Sea were generated by blooms of eukaryotic microalgae (i.e., diatoms which contain fucoxanthin), which reach the highest biomass peaks at the BATS station^39^. However, since Chl *a* and fucoxanthin are positively associated with NPP, the long-term productivity decrease could be explained by weakening of phytoplankton blooms during the investigated period. Lutein-zeaxanthin, which is diagnostic of *Synechococcus* cyanobacteria, also exerted a positive but smaller magnitude impact on NPP (100% positive sensitivity was gathered in four experiments only, Fig. 4B; Tables S4–13). Chl *b*, diagnostic of *Prochlorococcus* cyanobacteria, had no association with NPP.

### Ocean chemistry and productivity decrease

Our results are consistent with previous studies showing that both eukaryotic and prokaryotic microalgae drive phytoplankton biomass variability at the BATS site. Based on previous studies, the relative contributions from eukaryotes/prokaryotes can change in response to: (i) seasonal hydrodynamic modifications in the water column (production is generally higher during winter-spring), and (ii) shifts in the dominant circulation regime occurring in the Sargasso Sea over multi-annual time scales (i.e., relating to the North Atlantic Oscillation)^39^.

However, at both annual and multi-annual time scales, phytoplankton composition depends on the state of the chemical environment, i.e., which nutrients are present and at what concentrations and supply rates^13^. In this context, nutrient remineralization plays an important role in open-ocean productivity, by driving processes underlying NPP trends. Based on our ML analyses, nitrates are the only inorganic compound exerting a significant impact on primary production in the Sargasso Sea, while phosphates and silicates do not (the former never being significant and the latter showing a 100% positive sensitivity in only one experiment; see Fig. 4; Tables S4–13). Furthermore, since ML analysis was based on raw data points, we can reliably exclude that the analysis of the association between NPP and phosphates was affected by the single and isolated spike shown by the latter at the end of the time series (see Fig. 2A).

Thus, nitrates showed a significant positive impact on NPP (100% sensitivity) in five out of ten ML-experiments, and these nutrients were interrelated with pigments (they all had positive coefficients in the equations resulting from GP). This suggests that higher concentrations of nitrates were essential to drive blooms of phytoplankton groups having Chl *a* and fucoxanthin, such as diatoms. Thus, a decrease of nitrate supply to surface waters by the intensification of stratification would have reduced phytoplankton bloom magnitude in the photic zone. Conversely, the lack of influence of phosphates and silicates on NPP, revealed by GP results, could be explained as follows.

Firstly, phosphate is quickly re-mineralised and, therefore, made biologically available in the upper 50 m of the water column of the observed region^40^. This fast recycling would limit the negative impact exerted by the enhanced stratification on phosphate replenishment in the photic zone. In addition, changes in phytoplankton composition due to the relative increase of prokaryotes (see the higher values of Chl *b*, in comparison to Chl *a*, during the last decade of the BATS series; Fig. 2) can change phytoplankton C:P ratios to an extent that they need 50–67% less phosphates to maintain the same level of NPP^35^.

Secondly, silicates are essential for some eukaryotic microalgae to build exoskeletons, but concentrations in the upper 100 m of the Sargasso Sea are never depleted to levels observed for nitrates^19^. The surface ocean is under-saturated in silicate because the frustules of dead diatoms dissolve rapidly and release Si that is quickly assimilated by living diatom cells that require low concentrations to support vegetative growth^41,42^.

## Concluding Remarks

Our analyses of a 27-year oceanographic observational record show that a temperature increase of +0.021 °C per year was synchronous with a phytoplankton-productivity decrease of −5.6 mg C m^−2^ d^−1^ per year in the sub-tropical Sargasso Sea. These changes likely modified biogeochemical cycles at a regional scale and, eventually, induced important ecological changes in those marine systems^43^.

**Figure 5:**
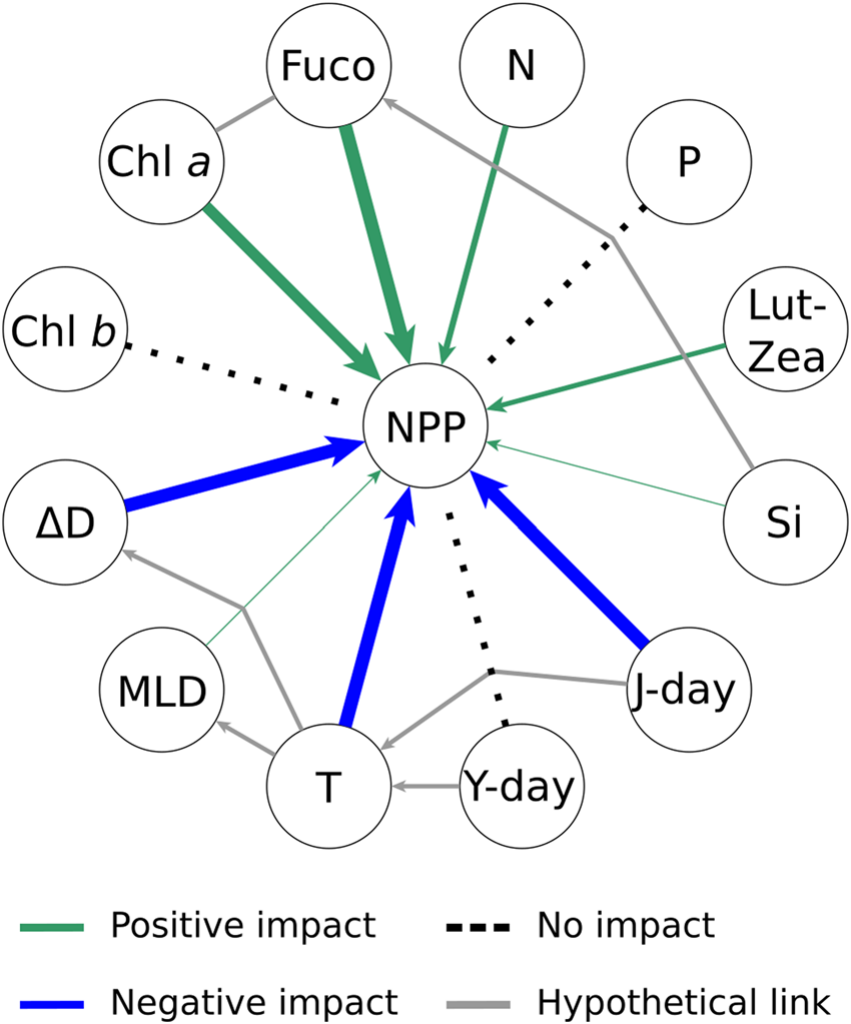
Systemic impact on net primary productivity at the BATS site. Overall impact of different variables (N = nitrates; P = phosphates; Lut-Zea = lutein-zeaxanthin; Si = silicates; J-day = Julian day; Y-day = the day of the year; T = temperature; MLD = mixed layer depth, ΔD = density gradient; Chl *b* = chlorophyll *b*; Chl *a* = chlorophyll *a*; Fuco = fucoxanthin) on net primary productivity NPP, according to the average of ten GP experiments shown in Fig. 4; green and blue arrows indicate either positive or negative impacts of a variable on NPP, respectively; arrow thickness is proportional to the magnitude of the impact.

Our analyses also explain why full understanding of NPP trends and their implications requires consideration of both ecological and biogeochemical processes, in addition to the state and dynamics of chemical and physical variables that have been the focus of most studies (Fig. 5).

Machine learning results suggest that pigments Chl *a* and fucoxanthin (indicators of community composition) act in synergy with nitrates (a potentially limiting factor) in driving NPP at BATS. However, silicates have a much weaker association and phosphates have no association with NPP changes. These observations can be explained by several factors, including:

(i) higher plasticity of phytoplankton cells in their P-storage than N-storage, which makes N a local nutrient control for all phytoplankton);
(ii) the quantitatively important, but poorly understood and measured, roles of dissolved organic N and P; and
(iii) a weak regulative role played by Si relative to other nutrients.

The ML-based associations between water temperature, stratification and primary productivity, plus other systemic properties such as nutrient concentrations and photosynthetic pigments in the Sargasso Sea at BATS station (Fig. 5) validate simulations with biogeochemical models that show a negative effect of global warming on phytoplankton activity through its influence on vertical circulation in the surface ocean^14,15^ (Fig. 1).

Finally, our results highlight the urgent need to couple long-term ocean monitoring with short-term measurements of key processes, eventually employing next-generation techniques like meta-omics, to strengthen our mechanistic understanding of past trends in order to forecast the state of future oceans^44,45^.

## Methods

### Data

Data used in this study were downloaded from http://bats.bios.edu/bats-data/. Methods pertaining to the sample collection and analysis and data production are available in^19^. The analyses presented herein considered data collected from 1990 to 2016.

Most of the variables we analysed were expressed as depth-integrated values. This choice was driven by the fact that we focused on NPP, which is a vector quantity – it can assume either positive or negative values (e.g., when respiration is higher than production) – and, by convention, it is more frequently expressed as an integrated value in oceanography. Among environmental variables, temperature is a scalar quantity and it is usually expressed as an average value. We followed this convention since these differences could not affect our mathematical tests, apart from constant factors.

### Long-term trend analysis

The analysis of long-term trends was performed from 1990 to 2016. Long-term trends were detected with the Seasonal Kendall test of monthly time series, implemented with the *seaKen* function in **R** package *wql* (a maintained version of now-archived package wq: http://cran.r-project.org/package=wq)^46,47^. Time-windowed analysis was carried out using the *seaRoll* function in **R** package *wql* with a window width of ten years. As regular monthly sampling of all variables at BATS did not begin until 1992 and the early data gaps could influence long-term trends, we interpolated missing data for the those very first years of the record. Data gaps were filled by interpolation using function i*nterpTs*, filling missing values with means for the corresponding month.

### Machine learning (ML): summary

We employed a procedure called ‘supervised machine learning’, in which a training set, i.e. a set of ‘known’ data composed of a list of rows in which x and y variables are both defined, is used to train the function. Knowing the correct answers, the algorithm iteratively makes predictions based on the training data and is corrected by making updates. Learning stops when the algorithm achieves an acceptable level of performance, which is assessed by means of a validation test called k-fold Cross-Validation^48,49^. The validation test estimates how the algorithm is expected to perform in general when used to make predictions using data not used during the training phase.

In all the ML experiments we performed, we searched for a formula *y* = *f(x)* satisfying the equation:

$$NPP=f(N,P,Lut \mbox{-} Zea,Si,J \mbox{-} day,Y \mbox{-} day,T,MLD,\Delta D,Chl\,{\rm{b}},Chl\,{\rm{a}},Fuco)$$

where *NPP* = net primary productivity (integrated values, between 0 and 120 m depths); *N* = nitrates (integrated, 0–120 m); *P* = phosphates (integrated, 0–120 m); *Lut-Zea* = lutein-zeaxanthin (integrated, 0–120 m); *Si* = silicates (integrated, 0–120 m); *J-day* = Julian day; *Y-day* = the day of the year, ordered from 1 to 365 (or 366, in leap years), i.e. a parameter used to associate a day of the year to the other variables taken in consideration and to express the progress of seasons; *T* = temperature (average, 0–120 m); *MLD* = mixed layer depth, *ΔD* = density gradient, as the ratio between densities at 20 and 120 m depts; *Chl* b = chlorophyll *b* (integrated, 0–120 m); *Chl* a = chlorophyll *a* (integrated, 0–120 m); *Fuco* = fucoxanthin (integrated, 0–120 m). All ML analyses were carried out using raw data but using only dates including values for each of the above-listed variables, thus excluding dates showing missing values.

In conducting ML, we employed mathematical techniques selected among the most commonly used in environmental studies, because of their power to identify plausible causative relationships between high-variance and inter-dependent variables^50,51^. The techniques applied herein were: (i) Gaussian Processes (Linear Kernel)^52^, (ii) Linear Regression Model^52^, (iii) Random Forest^53^, (iv) Support Vector Machine^52^, (v-vi) Multilayer Perceptron (automatic and manual modes)^50,52,54–56^, and (vii) Genetic Programming^24–26,57^. Techniques (i-v) (more details in Supporting methods) were applied by using the Waikato Environment for Knowledge Analysis (Weka), i.e. a comprehensive suite of Java class libraries that implement many state-of-the-art machine learning, data mining algorithms and data pre-processing tools, developed by the University of Waikato, New Zealand, and available as open source software^58^. Details for the analysis pertaining to Multilayer Perceptron (manual mode), and Genetic Programming are added in the following paragraphs. All the applied ML techniques were evaluated by a k-fold Cross-Validation.

### Validation of ML results

A k-fold Cross-Validation methodology was applied^48,49^. k-fold Cross-Validation is a data handling procedure used in machine learning to estimate generated predictive models (say, equations), i.e. in order to estimate how the model is expected to perform in general when used to make predictions on data not used during the training phase. k-fold Cross-Validation is a common method because it is easily applied and it shows a lower bias within all statistical evaluation methods^48,49^.

The general procedure is as follows:

1. Split the dataset into k disjoint groups of the same size;
2. For each unique group:
  I. Take the group as a hold out or validation data set;
  II. Take the remaining k-1 groups as a training data set;
  III. Fit a model on the training set and evaluate it on the validation set;
  IV. Retain the evaluation score and discard the model;
  V. Repeat the procedure from step I;
3. Summarize the skill of the model using the sample of model evaluation scores.

k is a parameter and represents how many subsets can be derived from an original dataset. Generally, we must be careful when choosing a value for k because we need to subdivide the dataset maintaining a good representation of the whole set. Otherwise we can occur in a high variance or bias that means we are overestimating the models. k = 5 or k = 10 would lead to models with limited bias and lower variance; for the present study we set k = 10.

### Multi-layer perceptron (manual mode)

This is a category of Artificial Neural Network (ANN)^52,54–56^. In the manual case the *ANN topology* (number of hidden layers, number of neurons in the hidden layers) was selected by a *pruning*/*growing* methodology^50^, starting from an initial random choice. The resulting topology was quite simple, and consisted of 8 input, one hidden layer – made up of 3 neurons - and one output. The *initial network weights Wt* were randomly chosen in a fixed range. The *learning rate*, a measure of the influence degree, in the formula for updating weights of the actual error, and the *momentum term*, that determines the influence of the history of weight changes, were determined by a trials-and-errors methodology. The training was made by the *back-propagation* procedure^55^. The number of epochs (training cycles) was dynamically determined by an early stopping criterion. The experiments were performed on the basis of the dataset described in the previous section, by using a neural network Excel-based simulation environment developed by Angshuman Saha (available at http://xoomer.virgilio.it/srampone/NNpred01.zip). The ANN settings are reported in Table S3.

### Genetic programming

Genetic Programming (GP)^24–26,57^ relied on a set of component functions, which include *arithmetic operators* (+, −, *, /), trigonometric functions (*sine*, *cosine and tangent and hyperbolic versions*) including their inverse, the *exponential* and the *natural logarithm*, the *logistic function*, and the *gauss function*. The function quality (*fitness measure*) was the Absolute Error. The GP experiments were performed by the genetic programming software tool *Eureqa*^59^, run for about 200,000 generations. Ten independent GP experiments were run (as replicates), resulting in ten distinct equations describing NPP in function of the other independent variables (see equations below):

1 $${\boldsymbol{y}}=7.114{{\rm{e}}}^{4}+0.06431\ast {{\boldsymbol{x}}}_{{\boldsymbol{9}}}+2.427{{\rm{e}}}^{-7}\ast {{{\boldsymbol{x}}}_{{\boldsymbol{11}}}}^{2}-0.003803\ast {{\boldsymbol{x}}}_{{\boldsymbol{11}}}-7.022{{\rm{e}}}^{4}\ast {{\boldsymbol{x}}}_{{\boldsymbol{4}}}-0.0009147\ast {{\boldsymbol{x}}}_{{\boldsymbol{1}}}\ast {{\boldsymbol{x}}}_{{\boldsymbol{5}}}$$

2 $${\boldsymbol{y}}=792.9+1.49\ast {{\boldsymbol{x}}}_{{\boldsymbol{6}}}+0.09097\ast {{\boldsymbol{x}}}_{{\boldsymbol{9}}}+4.264{{\rm{e}}}^{-6}\ast {{\boldsymbol{x}}}_{{\boldsymbol{12}}}\ast {{{\boldsymbol{x}}}_{{\boldsymbol{3}}}}^{2}-0.013\ast {{\boldsymbol{x}}}_{{\boldsymbol{1}}}-0.00675\ast {{\boldsymbol{x}}}_{{\boldsymbol{3}}}\ast {{{\boldsymbol{x}}}_{{\boldsymbol{5}}}}^{2}$$

3 $${\boldsymbol{y}}=8.915{{\rm{e}}}^{4}+0.05748\ast {{\boldsymbol{x}}}_{{\boldsymbol{9}}}+0.03729\ast {{\boldsymbol{x}}}_{{\boldsymbol{11}}}+1.383\ast {{\boldsymbol{x}}}_{{\boldsymbol{3}}}\ast \,\cos (19.8\ast {{\boldsymbol{x}}}_{{\boldsymbol{3}}})-61.88\ast {{\boldsymbol{x}}}_{{\boldsymbol{5}}}-8.773{{\rm{e}}}^{4}\ast {{\boldsymbol{x}}}_{{\boldsymbol{4}}}-8.336{{\rm{e}}}^{-7}\ast {{\boldsymbol{x}}}_{{\boldsymbol{1}}}\ast {{\boldsymbol{x}}}_{{\boldsymbol{11}}}$$

4 $${\boldsymbol{y}}=1.062{{\rm{e}}}^{5}+0.05927\ast {{\boldsymbol{x}}}_{{\boldsymbol{9}}}+0.009645\ast {{\boldsymbol{x}}}_{{\boldsymbol{11}}}-0.01879\ast {{\boldsymbol{x}}}_{{\boldsymbol{1}}}-47.86\ast {{\boldsymbol{x}}}_{{\boldsymbol{5}}}-1.044{{\rm{e}}}^{5}\ast {{\boldsymbol{x}}}_{{\boldsymbol{4}}}$$

5 $${\boldsymbol{y}}=1.196{{\rm{e}}}^{5}+0.0129\ast {{\boldsymbol{x}}}_{{\boldsymbol{11}}}-77.74\ast {{\boldsymbol{x}}}_{{\boldsymbol{5}}}-1.18{{\rm{e}}}^{5}\ast {{\boldsymbol{x}}}_{{\boldsymbol{4}}}$$

6 $${\boldsymbol{y}}=9.061{{\rm{e}}}^{4}+{{\boldsymbol{x}}}_{{\boldsymbol{6}}}+0.06108\ast {{\boldsymbol{x}}}_{{\boldsymbol{9}}}+0.008486\ast {{\boldsymbol{x}}}_{{\boldsymbol{11}}}-0.0126\ast {{\boldsymbol{x}}}_{{\boldsymbol{10}}}-0.01606\ast {{\boldsymbol{x}}}_{{\boldsymbol{1}}}-43.62\ast {{\boldsymbol{x}}}_{{\boldsymbol{5}}}-8.902{{\rm{e}}}^{4}\ast {{\boldsymbol{x}}}_{{\boldsymbol{4}}}$$

7 $${\boldsymbol{y}}=1.02{{\rm{e}}}^{5}+{{\boldsymbol{x}}}_{{\boldsymbol{6}}}+0.06454\ast {{\boldsymbol{x}}}_{{\boldsymbol{9}}}+0.03581\ast {{\boldsymbol{x}}}_{{\boldsymbol{12}}}-{{\boldsymbol{x}}}_{{\boldsymbol{3}}}\ast \,\sin (17.72\ast {{\boldsymbol{x}}}_{{\boldsymbol{11}}})-70.7\ast {{\boldsymbol{x}}}_{{\boldsymbol{5}}}-1.005{{\rm{e}}}^{5}\ast {{\boldsymbol{x}}}_{{\boldsymbol{4}}}$$

8 $${\boldsymbol{y}}=1{{\rm{e}}}^{5}+0.07668\ast {{\boldsymbol{x}}}_{{\boldsymbol{9}}}+0.04596\ast {{\boldsymbol{x}}}_{{\boldsymbol{1}}}+6.468{\rm{e}}-8\ast {{\boldsymbol{x}}}_{{\boldsymbol{6}}}\ast {{{\boldsymbol{x}}}_{{\boldsymbol{12}}}}^{2}-1.004{{\rm{e}}}^{5}\ast {{\boldsymbol{x}}}_{{\boldsymbol{4}}}-4.023{{\rm{e}}}^{-8}\ast {{\boldsymbol{x}}}_{{\boldsymbol{5}}}\ast {{{\boldsymbol{x}}}_{{\boldsymbol{1}}}}^{2}$$

9 $${\boldsymbol{y}}=9.307{{\rm{e}}}^{4}+0.05733\ast {{\boldsymbol{x}}}_{{\boldsymbol{9}}}+0.009636\ast {{\boldsymbol{x}}}_{{\boldsymbol{11}}}-{{\boldsymbol{x}}}_{{\boldsymbol{8}}}\ast \,\cos (0.05969\ast {{\boldsymbol{x}}}_{{\boldsymbol{9}}}\ast {{\boldsymbol{x}}}_{{\boldsymbol{11}}})-0.01275\ast {{\boldsymbol{x}}}_{{\boldsymbol{1}}}-54.09\ast {{\boldsymbol{x}}}_{{\boldsymbol{5}}}-9.143{{\rm{e}}}^{4}\ast {{\boldsymbol{x}}}_{{\boldsymbol{4}}}$$

10 $${\boldsymbol{y}}=978+{{\boldsymbol{x}}}_{{\boldsymbol{6}}}+0.06605\ast {{\boldsymbol{x}}}_{{\boldsymbol{9}}}+0.005877\ast {{\boldsymbol{x}}}_{{\boldsymbol{11}}}+57.83\ast \,\sin (1959\ast {{\boldsymbol{x}}}_{{\boldsymbol{9}}})+4.59{{\rm{e}}}^{-6}\ast {{\boldsymbol{x}}}_{{\boldsymbol{12}}}\ast {{{\boldsymbol{x}}}_{{\boldsymbol{3}}}}^{2}-0.01948\ast {{\boldsymbol{x}}}_{{\boldsymbol{1}}}-3.408\ast {{\boldsymbol{x}}}_{{\boldsymbol{3}}}$$

where, ***y*** is the net primary productivity integrated between 0 and 120 m depths, ***x***_***1***_ is the Julian day, ***x***_***2***_ is the day of the year, ***x***_***3***_ is the mixed layer depth, ***x***_***4***_ is the density gradient between 20 and 120 m depths, ***x***_***5***_ is the average temperature between 0 and 120 m depths, ***x***_***6***–***12***_ are the integrated values of nitrate, phosphate, silicate, fucoxanthin, chlorophyll *b*, chlorophyll *a*, and lutein + zeaxanthin, respectively, between 0 and 120 m depths (integrated values).

The relevance of each characteristic variable in determining the solution of the equation shown above was determined in terms of: (i) *Sensitivity*, i.e., the relative impact that the variable has on the solution result; (ii) % *Positive*, i.e., the likelihood that, by increasing the variable, the solution result will increase; (iii) *Positive Magnitude*, i.e., a measure of how big the positive impact of the variable is; (iv) % *Negative*, i.e., the likelihood that, by increasing this variable, the solution result will decrease; and (v) *Negative Magnitude*, a measure of how big the negative impact of the variable is. The synthetic results of sensitivity analyses are shown in Supporting information (Tables S4–14).

## Supplementary information

Supplementary information.

## Data Availability

Data used in this study are available at http://bats.bios.edu/bats-data/. Methods pertaining to the sample collection and analysis and data production are available in^19^.
