# Supplementary material for: Machine learning identifies a strong association between warming and reduced primary productivity in an oligotrophic ocean gyre
Source: Sci Rep. 2020 Feb 25;10:3287. doi: 10.1038/s41598-020-59989-y (PMC7042350; doi:10.1038/s41598-020-59989-y)
Supplement: Supplementary file 1 — Supplementary information. [file 41598_2020_59989_MOESM1_ESM.pdf]

Supplementary information

(Supplementary tables and methods)

Machine learning identifies a strong association between warming and reduced primary productivity in an oligotrophic ocean gyre

Domenico D'Alelio[*,§,1] , Salvatore Rampone[*,2], Luigi Maria Cusano[2], Valerio Morfino[2], Luca Russo[1], Nadia Sanseverino[2], James E. Cloern[3], Michael W. Lomas[§,4]

[*] Equal contribution

[§] Corresponding authors: domenico.dalelio@szn.it; mlomas@bigelow.org

[1] Stazione Zoologica Anton Dohrn, Department of Integrative Marine Ecology, Villa Comunale, I-80121, Naples, Italy

[2] Università degli Studi del Sannio, Via Traiano 3, I-82100, Benevento, Italy

[3] United States Geological Survey (emeritus), Menlo Park, CA, USA

[4] Bigelow Laboratory for Ocean Sciences, East Boothbay, ME, USA

Table S1. Long-term trends of physical, chemical, and biological properties in time series of monthly mean values from measurements made between 1990 and 2016. Trends are estimates of the Sen slope (a measure of the magnitude of a trend) and its significance (*p*) from the Seasonal Kendall test of monthly time series, implemented with the *seaKen* function in R package *wql* (a maintained version of now-archived package *wq*, available at: http://cran.r-project.org/package=wq).

Variable	Sen Slope	*p*
MLD	0.08	0.23
Density	0.00	0.93
Temperature	0.02	0.00
NO_3	0.26	0.06
PO_4	0.04	0.00
Si	-0.62	0.04
Chl *b*	42.87	0.04
Chl *a*	-11.41	0.82
NPP	-4.64	0.00

Table S2. Windowed trends of temperature and net primary production (NPP) over sequential decades beginning in 1990. Trends are estimates of the Sen slope and its significance (*p*) from time series of monthly mean values, using the *seaRoll* function in R package *wql* with a window width of ten years. Data gaps were filled by interpolation using function i*nterpTs*, filling missing values with means for the corresponding month. Statistically significant analyses are grey-marked.

Decade	Sen Slope Temperature	*p*		Sen Slope NPP	*p*
1990-1999	0.00	0.72		1.58	0.72
1991-2000	0.03	0.10		4.43	0.23
1992-2001	0.02	0.14		9.53	0.13
1993-2002	0.04	0.04		20.21	0.00
1994-2003	0.06	0.00		18.16	0.00
1995-2004	0.08	0.00		5.54	0.22
1996-2005	0.02	0.12		0.62	0.88
1997-2006	-0.01	0.54		4.19	0.57
1998-2007	-0.01	0.41		-2.69	0.50
1999-2008	0.00	0.86		-9.76	0.01
2000-2009	0.01	0.36		-10.91	0.01
2001-2010	0.00	1.00		-11.80	0.01
2002-2011	-0.03	0.13		-8.71	0.05
2003-2012	-0.01	0.39		-5.47	0.20
2004-2013	0.00	0.76		-5.28	0.20
2005-2014	0.01	0.29		-9.44	0.01
2006-2015	0.02	0.10		-12.70	0.00
2007-2016	0.03	0.02		-15.95	0.00

Table S3. Adjustable Parameters of the Excel-based system which simulates the MLP neural network.

Network Architecture Options					
**Number of Inputs**	**8**	Number of Outputs		**1**	
**Number of Hidden Layers**	**1**	Hidden Layer sizes		**Hidden1**<br>**3**	**Hidden2**<br>**0**
**Learning parameter *(between 0 and 1)***	**0.4**	Initial W_t Range (0 +/- w): w =		**0.5**	
**Momentum *(between 0 and 1)***	**0**				
**Total #Labelled Feature Vectors**	**122**	No. of Training cycles		**500**	
**Present Inputs in Random order while Training *(YES or NO)***	**NO**	Training Mode *(Batch or Sequential)*		**Sequential**	
**Save Network weights**	**With least Validation Error**				
**Training / Validation Set**	**Partition data into Training / Validation set**				
**How the Validation set is selected *(1 or 2)***	**2**	**Option 1 : Randomly**	10%	**of data as Validation set**	
		**Option 2: Use last**	12	**rows of the data as validation set**	

Table S4. Sensitivity analyses for GP equation #1.

Variable	Sensitivity	% Positive	Positive Magnitude	% Negative	Negative Magnitude
x_4	0.49891	0%	0	100%	0.49891
x_5	0.47887	0%	0	100%	0.47887
x_{11}	0.41818	99%	0.42094	1%	0.049305
x_1	0.33796	0%	0	100%	0.33796
x_9	0.29622	100%	0.29622	0%	0

x_1 is the Julian day, x_2 is the day of the year, x_3 is the mixed layer depth, x_4 is the density gradient between 20 and 120 m depths, x_5 is the average temperature between 0 and 120 m depths, $x_{6\text{-}12}$ are the integral values of nitrate, phosphate, silicate, fucoxanthin, chlorophyll *b*, chlorophyll *a*, and lutein + zeaxanthin, respectively, between 0 and 120 m depths.

Table S5. Sensitivity analyses for GP equation #2.

Variable	Sensitivity	% Positive	Positive Magnitude	% Negative	Negative Magnitude
x_3	0.64582	23%	0.68279	77%	0.63459
x_9	0.41163	100%	0.41163	0%	0
x_6	0.2903	100%	0.2903	0%	0
x_{12}	0.24487	100%	0.24487	0%	0
x_5	0.23268	0%	0	100%	0.23268
x_1	0.21699	0%	0	100%	0.21699

x_1 is the Julian day, x_2 is the day of the year, x_3 is the mixed layer depth, x_4 is the density gradient between 20 and 120 m depths, x_5 is the average temperature between 0 and 120 m depths, $x_{6\text{-}12}$ are the integral values of nitrate, phosphate, silicate, fucoxanthin, chlorophyll *b*, chlorophyll *a*, and lutein + zeaxanthin, respectively, between 0 and 120 m depths.

Table S6. Sensitivity analyses for GP equation #3.

Variable	Sensitivity	% Positive	Positive Magnitude	% Negative	Negative Magnitude
x_3	30.295	50%	33.892	50%	26.768
x_5	0.78441	0%	0	100%	0.78441
x_4	0.59281	0%	0	100%	0.59281
x_1	0.2844	0%	0	100%	0.2844
x_{11}	0.28197	100%	0.28197	0%	0
x_9	0.25182	100%	0.25182	0%	0

x_1 is the Julian day, x_2 is the day of the year, x_3 is the mixed layer depth, x_4 is the density gradient between 20 and 120 m depths, x_5 is the average temperature between 0 and 120 m depths, $x_{6\text{-}12}$ are the integral values of nitrate, phosphate, silicate, fucoxanthin, chlorophyll *b*, chlorophyll *a*, and lutein + zeaxanthin, respectively, between 0 and 120 m depths.

Table S7. Sensitivity analyses for GP equation #4.

Variable	Sensitivity	% Positive	Positive Magnitude	% Negative	Negative Magnitude
x_4	0.69539	0%	0	100%	0.69539
x_5	0.59779	0%	0	100%	0.59779
x_{11}	0.5891	100%	0.5891	0%	0
x_1	0.29929	0%	0	100%	0.29929
x_9	0.25585	100%	0.25585	0%	0

x_1 is the Julian day, x_2 is the day of the year, x_3 is the mixed layer depth, x_4 is the density gradient between 20 and 120 m depths, x_5 is the average temperature between 0 and 120 m depths, $x_{6\text{-}12}$ are the integral values of nitrate, phosphate, silicate, fucoxanthin, chlorophyll *b*, chlorophyll *a*, and lutein + zeaxanthin, respectively, between 0 and 120 m depths.

Table S8. Sensitivity analyses for GP equation #5.

Variable	Sensitivity	% Positive	Positive Magnitude	% Negative	Negative Magnitude
x_5	0.9383	0%	0	100%	0.9383
x_{11}	0.7616	100%	0.7616	0%	0
x_4	0.75897	0%	0	100%	0.75897

x_1 is the Julian day, x_2 is the day of the year, x_3 is the mixed layer depth, x_4 is the density gradient between 20 and 120 m depths, x_5 is the average temperature between 0 and 120 m depths, $x_{6\text{-}12}$ are the integral values of nitrate, phosphate, silicate, fucoxanthin, chlorophyll *b*, chlorophyll *a*, and lutein + zeaxanthin, respectively, between 0 and 120 m depths.

Table S9. Sensitivity analyses for GP equation #6.

Variable	Sensitivity	% Positive	Positive Magnitude	% Negative	Negative Magnitude
x_4	0.62909	0%	0	100%	0.62909
x_5	0.57825	0%	0	100%	0.57825
x_{11}	0.55011	100%	0.55011	0%	0
x_9	0.27983	100%	0.27983	0%	0
x_1	0.27154	0%	0	100%	0.27154
x_{10}	0.21141	0%	0	100%	0.21141
x_6	0.19726	100%	0.19726	0%	0

x_1 is the Julian day, x_2 is the day of the year, x_3 is the mixed layer depth, x_4 is the density gradient between 20 and 120 m depths, x_5 is the average temperature between 0 and 120 m depths, $x_{6\text{-}12}$ are the integral values of nitrate, phosphate, silicate, fucoxanthin, chlorophyll *b*, chlorophyll *a*, and lutein + zeaxanthin, respectively, between 0 and 120 m depths.

Table S10. Sensitivity analyses for GP equation #7.

Variable	Sensitivity	% Positive	Positive Magnitude	% Negative	Negative Magnitude
x_{11}	102.57	52%	121.55	48%	82.129
x_5	0.98137	0%	0	100%	0.98137
x_4	0.74366	0%	0	100%	0.74366
x_{12}	0.34956	100%	0.34956	0%	0
x_9	0.30958	100%	0.30958	0%	0
x_6	0.20654	100%	0.20654	0%	0
x_1	0.021355	100%	0.021355	0%	0

x_1 is the Julian day, x_2 is the day of the year, x_3 is the mixed layer depth, x_4 is the density gradient between 20 and 120 m depths, x_5 is the average temperature between 0 and 120 m depths, $x_{6\text{-}12}$ are the integral values of nitrate, phosphate, silicate, fucoxanthin, chlorophyll *b*, chlorophyll *a*, and lutein + zeaxanthin, respectively, between 0 and 120 m depths.

Table S11. Sensitivity analyses for GP equation #8.

Variable	Sensitivity	% Positive	Positive Magnitude	% Negative	Negative Magnitude
x_5	0.90486	0%	0	100%	0.90486
x_4	0.77778	0%	0	100%	0.77778
x_1	0.42203	0%	0	100%	0.42203
x_9	0.38517	100%	0.38517	0%	0
x_6	0.2425	100%	0.2425	0%	0
x_{12}	0.14474	100%	0.14474	0%	0

x_1 is the Julian day, x_2 is the day of the year, x_3 is the mixed layer depth, x_4 is the density gradient between 20 and 120 m depths, x_5 is the average temperature between 0 and 120 m depths, $x_{6\text{-}12}$ are the integral values of nitrate, phosphate, silicate, fucoxanthin, chlorophyll *b*, chlorophyll *a*, and lutein + zeaxanthin, respectively, between 0 and 120 m depths.

Table S12. Sensitivity analyses for GP equation #9.

Variable	Sensitivity	% Positive	Positive Magnitude	% Negative	Negative Magnitude
x_9	176.15	53%	231.8	47%	114.41
x_{11}	35.75	60%	3.8731	40%	83.566
x_5	0.62348	0%	0	100%	0.62348
x_4	0.56179	0%	0	100%	0.56179
x_1	0.18734	0%	0	100%	0.18734
x_8	0.0087738	100%	0.0087738	0%	0

x_1 is the Julian day, x_2 is the day of the year, x_3 is the mixed layer depth, x_4 is the density gradient between 20 and 120 m depths, x_5 is the average temperature between 0 and 120 m depths, $x_{6\text{-}12}$ are the integral values of nitrate, phosphate, silicate, fucoxanthin, chlorophyll *b*, chlorophyll *a*, and lutein + zeaxanthin, respectively, between 0 and 120 m depths.

Table S13. Sensitivity analyses for GP equation #10.

Variable	Sensitivity	% Positive	Positive Magnitude	% Negative	Negative Magnitude
x_9	4376.4	52%	383.92	48%	8676
x_3	0.61066	24%	0.6432	76%	0.60022
x_{11}	0.33494	100%	0.33494	0%	0
x_1	0.28948	0%	0	100%	0.28948
x_{12}	0.23464	100%	0.23464	0%	0
x_1	0.17342	100%	0.17342	0%	0

x_1 is the Julian day, x_2 is the day of the year, x_3 is the mixed layer depth, x_4 is the density gradient between 20 and 120 m depths, x_5 is the average temperature between 0 and 120 m depths, $x_{6\text{-}12}$ are the integral values of nitrate, phosphate, silicate, fucoxanthin, chlorophyll *b*, chlorophyll *a*, and lutein + zeaxanthin, respectively, between 0 and 120 m depths.

Table S14. Synthesis of sensitivity analyses

Exp.	x1<br>J-day	x2<br>Y-day	x3<br>MLD	x4<br>ΔD	x5<br>T	x6<br>N	x7<br>P	x8<br>Si	x9<br>Fuco	x10<br>Chl b	x11<br>Chl a	x12<br>Lu-Ze
(1)	-1	0	0	-1	-1	0	0	0	1	0	1	0
(2)	-1	0	-0,8	0	-1	1	0	0	1	0	0	1
(3)	-1	0	0,5	-1	-1	0	0	0	1	0	1	0
(4)	-1	0	0	-1	-1	0	0	0	1	0	1	0
(5)	0	0	0	-1	-1	0	0	0	0	0	1	0
(6)	-1	0	0	-1	-1	1	0	0	1	0	1	0
(7)	0	0	0	-1	-1	1	0	0	1	0	0,5	1
(8)	-1	0	0	-1	-1	1	0	0	1	0	0	1
(9)	-1	0	0	-1	-1	0	0	1	0,5	0	0,6	0
(10)	-1	0	-0,8	0	0	1	0	0	0,5	0	1	1
Mean impact of the variable on NPP												
	**-80%**	0%	15%	**-80%**	**-90%**	50%	0%	10%	**80%**	0%	**71%**	40%

1 = positive impact, -1 = negative impact, 0 = no impact. x_1 is the Julian day, x_2 is the day of the year, x_3 is the mixed layer depth, x_4 is the density gradient between 20 and 120 m depths, x_5 is the average temperature between 0 and 120 m depths, x_{6-12} are the integral values of nitrate, phosphate, silicate, fucoxanthin, chlorophyll *b*, chlorophyll *a*, and lutein + zeaxanthin, respectively, between 0 and 120 m depths.

Supplementary methods

Gaussian Processes (Linear Kernel) – In the Gaussian Processes[1], the prediction is probabilistic (Gaussian) so that one can compute empirical confidence intervals and decide based on those if one should refit (online fitting, adaptive fitting) the prediction in some region of interest. Their greatest practical advantage is that they can give a reliable estimate of their own uncertainty. Since Gaussian processes let us describe probability distributions over functions, we can use Bayes' rule to update our distribution of functions by observing training data.

Linear Regression models – In this ML technique[1], a target prediction value based on independent variables is employed. It finds out a linear relationship between input and output.

Linear Random Forest – The Random Forest[2] is an evolution of the Decision Tree method. In a Decision Tree, all possible outcomes of a decision are shown using a tree branching methodology. The internal nodes are tests on various attributes, the branches of the tree are the outcomes of the tests and the leaf nodes are the decision made after computing all of the attributes. The Random Forests Algorithm handles some of the limitations of Decision Trees Algorithm, namely that the accuracy of the outcome decreases when the number of decisions in the tree increases. So, in the Random Forests Algorithm, there are multiple decision trees that represent various statistical probabilities. All of these trees are mapped to a single tree known as the CART model (Classification and Regression Trees). In the end, the final prediction for the Random Forests Algorithm is obtained by polling the results of all the decision trees.

Support Vector Machine – This is a machine learning tool for classification and regression[3]. Given labelled training data, the algorithm outputs an optimal hyperplane which categorizes new examples. In the case of regression, a margin of tolerance (epsilon) is set, individualizing the hyperplane which maximizes the margin.

1. Bishop, C. M. *Pattern recognition and machine learning*. (springer, 2006).
2. Breiman, L. Random forests. *Mach. Learn.* **45**, 5–32 (2001).
3. Scholkopf, B. & Smola, A. J. *Learning with kernels: support vector machines, regularization, optimization, and beyond*. (MIT press, 2001).
